# Could ultrasound-guided internal jugular vein catheter insertion replace the use of chest X-ray?

**DOI:** 10.1186/s13054-018-2130-x

**Published:** 2018-08-16

**Authors:** William F. Amaya-Zuñiga, Fernando Raffán-Sanabria

**Affiliations:** 1 Department of Anesthesia and Perioperative Medicine, Cardiovascular and Transplant Anesthesiologist, Hospital Universitario Fundación Santa Fe de Bogotá, Universidad de los Andes, Universidad el Bosque, Cll 119 # 7-75, Bogotá, Colombia; 2 Department of Anesthesia and Perioperative Medicine, Intensive Care Medicine and Transplant Anesthesiologist, Hospital Universitario Fundación Santa Fe de Bogotá, Universidad de los Andes, Universidad el Bosque, Bogotá, Colombia

The implementation of ultrasound protocols during catheter placement has demonstrated multiple advantages that increase accuracy and allow medical teams to reduce operative time, potential complications, and procedure costs [1, 2]. The wide use of lung and cardiac ultrasound in perioperative medicine enables earlier diagnosis of some acute complications, allowing prompt intervention and control of damage.

**Fig. 1:**
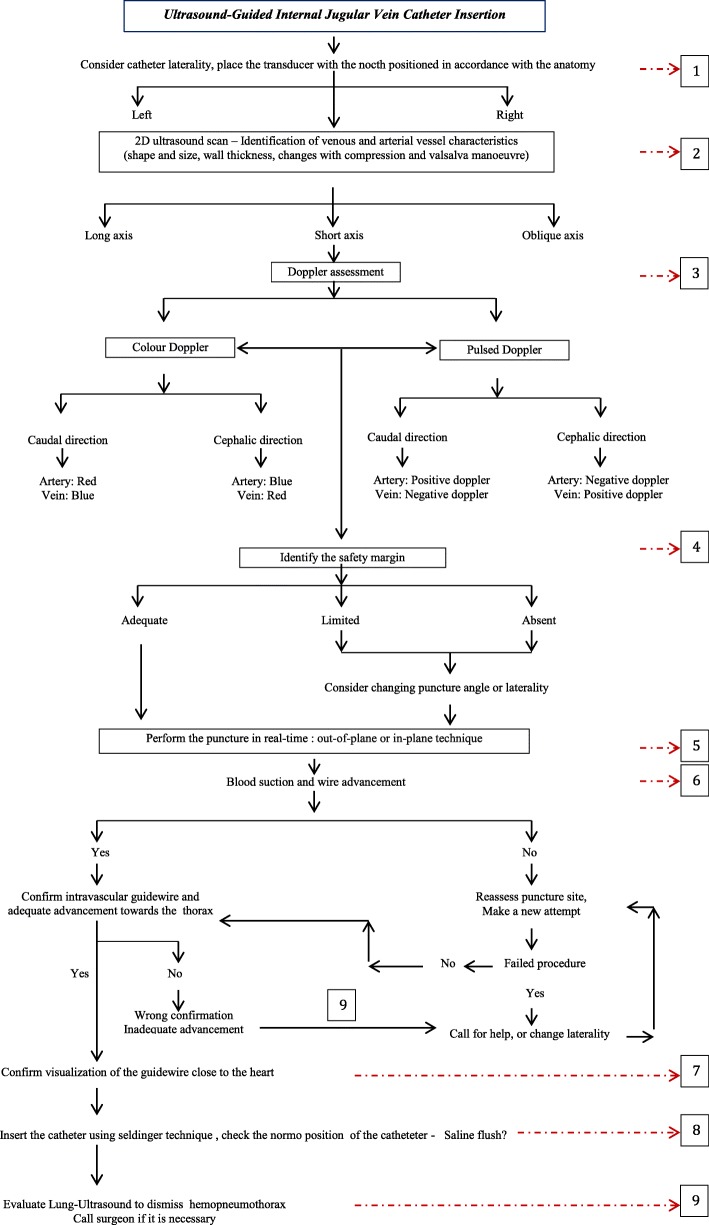
Ultrasound-Guided Internal Jugular Vein Catheter Insertion algorithm

We present the Successful Ultrasound-Guided Internal Jugular Vein Catheter Insertion algorithm (Fig. 1), which is based on reports of safe vascular ultrasound protocols in daily practice [3, 4]. It follows an operational pipeline that integrates steps focused on adequate vascular identification, cannulation, and placement of the catheter. The operational steps are:

- 1, 2 - A 2D vascular ultrasound in the Trendelenburg position identifies vessel characteristics and determines the “margin of safety”. If an overlap of the artery is present, there is a high possibility of artery injury.
- 3-Assessment of color Doppler and pulsed Doppler completes the vascular identification, which can differentiate their flows and discount the presence of thrombus.
- 4, 5 – Evaluation of a safety margin could guide modifications of puncture angles, avoiding injuries of the anterior or lateral artery wall.
- 6-After getting venous return, guidewire advancement should be smooth and unobstructed and the thoracic direction needs to be confirmed. The presence of intraluminal dissection or false pathways should be detected.
- 7, 8 - In the subcostal echocardiogram view, guidewire and catheter identification close to the heart helps to optimize adequate placement. The saline flush confirmation test can help to confirm this, but it should not be performed routinely.
- 9- Although the use of ultrasound in real time for central venous access reduces the number of pleuropulmonary complications, a lung ultrasound assessment should be performed, seeking to identify potential associated lesions, guaranteeing prompt attention to the patient.

The application of this algorithm optimizes the use of bedside ultrasound as a diagnostic tool, organizes the steps, and increases the early identification of potential operational errors. Rapid interventions significantly reduce the clinical impact on the patient and avoid the use of chest X-ray. Limitations for its implementation include the availability of the equipment with probes and staff training. This algorithm could also be useful in subclavian venous catheter placement, although the evidence does not statistically support the use of ultrasound for this application.
